# Additivity of Feature-Based and Symmetry-Based Grouping Effects in Multiple Object Tracking

**DOI:** 10.3389/fpsyg.2016.00657

**Published:** 2016-05-04

**Authors:** Chundi Wang, Xuemin Zhang, Yongna Li, Chuang Lyu

**Affiliations:** ^1^Beijing Key Lab of Applied Experimental Psychology, School of Psychology, Beijing Normal UniversityBeijing, China; ^2^State Key Laboratory of Cognitive Neuroscience and Learning and IDG/McGovern Institute for Brain Research, Beijing Normal UniversityBeijing, China; ^3^Center for Collaboration and Innovation in Brain and Learning Sciences, Beijing Normal UniversityBeijing, China; ^4^Department of Psychology, RenMin University of ChinaBeijing, China

**Keywords:** multiple object tracking, additivity, grouping, spatial-motion-based symmetry, feature-based tracking

## Abstract

Multiple object tracking (MOT) is an attentional process wherein people track several moving targets among several distractors. Symmetry, an important indicator of regularity, is a general spatial pattern observed in natural and artificial scenes. According to the “laws of perceptual organization” proposed by Gestalt psychologists, regularity is a principle of perceptual grouping, such as similarity and closure. A great deal of research reported that feature-based similarity grouping (e.g., grouping based on color, size, or shape) among targets in MOT tasks can improve tracking performance. However, no additive feature-based grouping effects have been reported where the tracking objects had two or more features. “Additive effect” refers to a greater grouping effect produced by grouping based on multiple cues instead of one cue. Can spatial symmetry produce a similar grouping effect similar to that of feature similarity in MOT tasks? Are the grouping effects based on symmetry and feature similarity additive? This study includes four experiments to address these questions. The results of Experiments 1 and 2 demonstrated the automatic symmetry-based grouping effects. More importantly, an additive grouping effect of symmetry and feature similarity was observed in Experiments 3 and 4. Our findings indicate that symmetry can produce an enhanced grouping effect in MOT and facilitate the grouping effect based on color or shape similarity. The “where” and “what” pathways might have played an important role in the additive grouping effect.

## Introduction

Multiple object tracking (MOT) is a tracking process involving the consumption of continuous attentional resources. In a typical MOT task, a number of identical objects are shown within a display. A subset is designated as “targets” through their flashing; thereafter, all objects move randomly around the display for several seconds. The task of the observer is to keep track of the target subset. At the end of the trial, the observer is required to indicate all the targets using a mouse (Pylyshyn, 2001). In a dynamic display, such as MOT, access must be maintained by the continued existence of objects, which is referred to as “objecthood” (Scholl and Pylyshyn, 1999; Blaser et al., 2000; Scholl, 2001; Scholl et al., 2001). According to Pylyshyn and Storm (1988), the maximum number of targets that can be tracked is five. The number of tracked targets is constrained by the limit of “tags” that the visual system can process simultaneously (Pylyshyn, 1989, 1994, 2001; Pylyshyn et al., 1994; Burkell and Pylyshyn, 1997). The “tagged” objects can be tracked successfully and can have an advantage in further processes (Pylyshyn, 1989, 1994, 2001; Pylyshyn et al., 1994; Burkell and Pylyshyn, 1997; Sears and Pylyshyn, 2000). The maximum number of “tags” is relatively fixed without practice and training (Pylyshyn, 1989; Burkell and Pylyshyn, 1997).

Grouping is a process of rearranging elements into larger units (Feldman, 1999), and is extensively considered to be automatic and preattentive (Prinzmetal and Banks, 1977; Kahneman and Henik, 1981; Treisman, 1982). However, there is evidence that suggesting the opposite. Treisman and Schmidt (1982) have shown that the binding of features into an object required attention. Yantis (1992) demonstrated two phases of grouping involved in MOT task: (1) the “stimulus-driven” and automatic “group formation” performed during target designation and (2) the “goal-directed” and effortful “group maintenance” performed during the tracking of the moving targets. Objects can be perceptually grouped based on different properties, such as regularity, connectivity, similarity, common fate, and contour interpolation. These groupings can be classified into two categories: one for clusters of grouped objects, such as common fate and similarity, and the other for the binding of fragments into a qualitatively different object, such as contour interpolation and connectivity. Previous studies (Yantis, 1992; Scholl et al., 2001; Suganuma and Yokosawa, 2006; Makovski and Jiang, 2009a,b; Keane et al., 2011; Feria, 2012; Howe and Holcombe, 2012; Erlikhman et al., 2013) have examined grouping based on virtual polygons (Yantis, 1992), connectivity (Scholl et al., 2001), interpolation (Keane et al., 2011), common fate (Yantis, 1992; Suganuma and Yokosawa, 2006), and similarity (Makovski and Jiang, 2009a,b; Feria, 2012; Howe and Holcombe, 2012; Erlikhman et al., 2013). These groupings in MOT are revealed by object-based, spatiotemporal and featural properties.

A few studies have reported that grouping in MOT can be object-based. Yantis (1992) has found that the formation and maintenance of coherent but nonrigid virtual polygons composed of target elements affect tracking performance. In addition, the degrees to which targets and distractors are connected affect tracking performance (Scholl et al., 2001); in that study, the authors used a single line or convex hull to connect a target and a distractor. With the merging of more targets and distracters, independently selecting and tracking the targets has become more difficult over time (i.e., the target-distractor grouping impaired tracking performance). In addition, Keane et al. (2011) showed that contour interpolation could automatically bind targets together to improve tracking performance in a multiple vertex tracking (MVT) tasks.

There is evidence that participants perform tracking on the basis of spatiotemporal properties. Researchers (e.g., Yantis, 1992; Suganuma and Yokosawa, 2006; Ericson and Beck, 2013) have found that people use information pertaining to motion, direction, and trajectory during tracking. The motion and direction information is represented in the visual memory in the same manner as object features, such as color (Stone, 1998, 1999; Newell et al., 2004; Horowitz and Cohen, 2010). Participants could maintain information about target location and motion when tracking multiple objects (Fencsik et al., 2007) and, in the process, learn the trajectories of attended objects (Makovski et al., 2008). An unexpected change of target trajectory impairs tracking performance (Ericson and Beck, 2013). Yantis (1992) found that a relative difference in velocity between targets and distractors enhances tracking performance, even if the velocity difference could not be consciously perceived. Suganuma and Yokosawa (2006) demonstrated that the tracking performance is impaired when targets and distractors are chasing one another or moving in the same direction. These studies suggest that spatiotemporal properties can be used during object tracking.

Many previous studies have shown that subjects track objects on the basis of featural properties (Horowitz et al., 2007; Makovski and Jiang, 2009a; Feria, 2012; Howe and Holcombe, 2012; Erlikhman et al., 2013). Makovski and Jiang (2009a) found that when objects have a unique color, their performance is generally improved compared with a homogenous condition. Feria (2012) found that distractors that are different from the targets in shape, color, or motion have a less intense effect on tracking compared with distractors sharing identical properties with the targets. Erlikhman et al. (2013) demonstrated that participants automatically use the physical features of objects for perceptual grouping. They tested the influence of eight features on tracking including color, contrast polarity, orientation, size, shape, combination (color, size, and shape), stereo depth, and interpolation. For each feature type they compared the target-distractor grouping (TDG) condition with a target-target grouping (TG) condition, a homogeneous condition and a diversity condition. The TDG condition was target-distractor grouping based on feature similarity, in which two targets and two distractors shared one feature (i.e., same color), and the remaining objects shared the other feature. The TG condition was inter-target grouping based on feature similarity, where targets all shared one feature and all distractors shared another. There were no target-target grouping or target-distractor grouping in the homogeneous condition and the diversity condition. They found that in the color, size, shape, combination, and interpolation conditions, tracking performance is improved for the target-target grouping and then impaired for the target-distractor grouping. Their results showed that when targets are grouped together automatically based on the same feature tracking performance can be significantly improved (Erlikhman et al., 2013). Considered as a whole, these studies suggest that featural properties can be used during tracking and that grouping in MOT can be feature-based.

Erlikhman et al. (2013) found that grouping features did not seem to be additive. They found that the magnitude of the TG/TDG difference for the combination of shape, color, and size do not surpass that of the individual features that composed it. Grouping based on color, shape, and size did not improve tracking performance more compared with grouping based on single color. In other words, when two different shapes were added to a condition where targets and distractors already had two different colors, performance was not improved compared with the previous condition. No greater grouping effect has been produced by multiple surface feature cues relative to a single feature cue. The authors also speculated that the visual system uses only the most salient surface feature for grouping (Erlikhman et al., 2013). In addition, Feria (2012) found that distractors that are different from the targets in two features impair tracking less compared with distractors that differ from the targets in only a single feature; however, the effect of the number of shared features occurs only when the tracking load is low. The abovementioned studies show that groupings provided by a single surface-feature source are not additive.

Previous studies on MOT (e.g., Erlikhman et al., 2013) have failed to find the additivity of grouping effects possibly because of the top-down processing of object features. According to Feria (2012), the top-down processing in MOT focuses on the conjunction of the targets' features but not on any one specific feature. In addition, no additive feature-based grouping effects are reported possibly because the grouping of color, shape, and size are based on the same basic grouping principle of similarity. Different grouping principles can either cooperate with one another to support the same perceptual grouping or compete with one another to support different perceptual groupings (Lier and Wagemans, 1997). Cooperating with one another offers a chance to study the additive grouping effect, whereas competing with one another offers the possibility of studying and identifying the stronger grouping principle.

In this study, we examine whether the grouping effects based on different principles (regularity and similarity) and properties (featural property and spatiotemporal property) are additive. We focus on two powerful grouping principles that have been attracting intensive research: regularity and similarity. We choose symmetry to offer spatiotemporal property and represent the regularity principle, and we choose color (or shape) to offer featural property and as a stand in for the similarity principle. Because symmetry-based and feature-based groupings are based on independent principles and properties, we predicted that there would be no interaction. To our knowledge, no study has demonstrated the automatic symmetry-based grouping effect in MOT. Therefore, our first aim is to test the symmetry-based grouping effect. Thereafter, we focus on exploring the additivity of feature-based and symmetry-based grouping effects.

**Figure 1:**
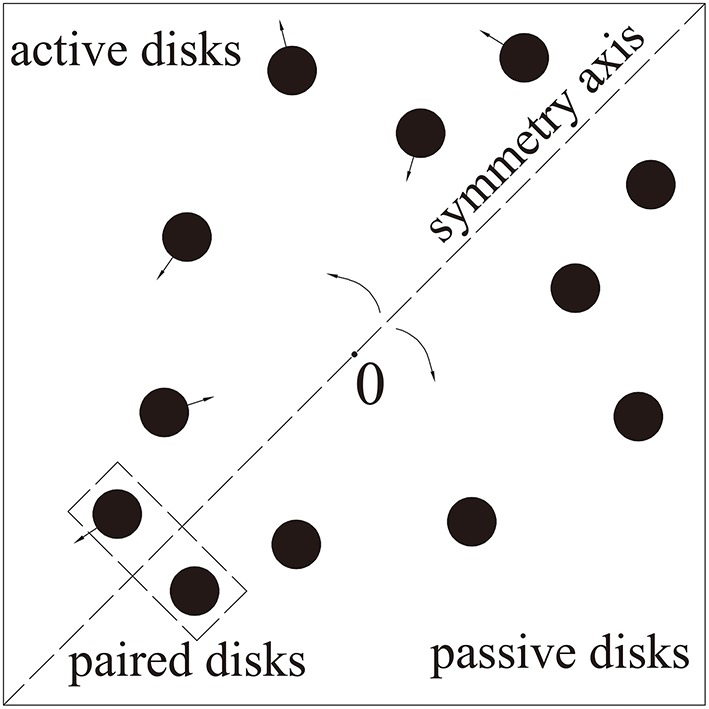
**Stimuli in SMOT**. The invisible axis rotated around the center of the presentation window. The starting positions and directions of the active disks were random. Each active disk was symmetrical with a passive disk. Coordinates of the passive disks were determined by coordinates of the active disks and the axis of symmetry. The disks bounced off each other, the edges of the presentation window and the invisible axis of symmetry.

Symmetry is an indicator of spatial regularity, which can be used to guide visual attention (Wagemans, 1993; Roggeveen et al., 2004). Wagemans (1993) showed that symmetry can be used to perceive visual forms, and symmetric patterns are more easily perceived than random patterns. Roggeveen et al. (2004) indicated that target-distractor symmetry affects search performance in the condition where grouping is based on inter-item shape symmetry. To test the symmetry-based grouping effect in dynamic scenes, we must modify the original MOT task to create a symmetric MOT (SMOT) task (see Figure 1). In SMOT, the items do not move independently around the display; instead, they are paired and presented symmetrically about the same invisible axis of symmetry. The axis of symmetry passes through and rotates around the center of the display. Objects located on one side of the invisible axis are active objects that move randomly during tracking. Meanwhile, objects located on the other side of the axis are passive objects whose locations are determined by the corresponding active objects and the axis of symmetry.

Using SMOT, we can test the symmetry-based grouping effect. Two grouping relations are involved in SMOT: target-target grouping (target and target are in a symmetric relation) and target-distractor grouping (target and distractor are in a symmetric relation). This dynamic symmetry-based grouping provides both spatial and motion information during tracking. A control condition is needed in testing whether symmetry-based grouping is automatic. Grouping is automatic if it occurred even when it is consistently detrimental to performance and irrelevant to task instructions. If the symmetry-based grouping automatically guides attention, better tracking performances are expected in the target-target grouping condition relative to the control condition and in the control condition relative to the target-distractor grouping condition.

Experiments 1 and 2 were conducted to examine the effect of automatic symmetry-based grouping on tracking performance. In Experiment 1, three conditions of grouping relations were designed: Symmetry (target-target grouping), Asymmetry (target-distractor grouping), and Random trajectory (control condition: no grouping relation). The control condition in Experiment 1 was designed to retain the trajectories of SMOT but not the paired symmetric motion of disks. Consequently, no grouping relation was formed based on symmetry in the Random trajectory condition. We also manipulated the target number in Experiment 1. Target number is an important factor that affects MOT performance, so it should be tested in the newly designed SMOT. In Experiment 2, a new control condition was designed to offer stronger evidence for automatic symmetry-based grouping. The new control condition owned a replication of the axis of symmetry of SMOT, which retained all the motion parameters of the axis of symmetry. Previous studies (Yantis, 1992; Suganuma and Yokosawa, 2006) have reported that spatiotemporal properties can be used to group objects during tracking. We predicted that symmetry would affect tracking performance. Furthermore, a previous study involving a visual search task has shown that spatial memory is modulated more by grouping based on spatial features than by grouping based on surface features of the presented objects (Conci and von Mühlenen, 2011). Therefore, a larger effect of symmetry than of similarity would result when they compete with one another to support different perceptual groupings.

In Experiments 3 and 4, the additivity of feature-based and symmetry-based grouping effects was explored. In Experiment 3, two grouping relations were based on symmetry: Symmetry (target-target grouping based on symmetry) and Asymmetry (target-distractor grouping based on symmetry). Moreover, three grouping relations were created based on color: Identical (no grouping relation), T-T (target-target grouping based on color), and T-D (target-distractor grouping based on color). Both types of symmetric and featural information were irrelevant to the task. To provide evidence for additivity, we compared the T-T Symmetry condition with the Identical Symmetry condition and with the T-T Asymmetry condition. Higher accuracy in the T-T Symmetry condition relative to the Identical Symmetry condition and to the T-T Asymmetry condition would be ascribed to the additivity of symmetry-based and color-based grouping effects. To add stronger and more reliable evidence for additivity, symmetry-based and shape-based grouping effects were tested in Experiment 4. The manipulations and predictions in Experiment 4 were similar to those in Experiment 3, except that the similarity grouping was shape-based.

## Experiment 1

**Figure 2:**
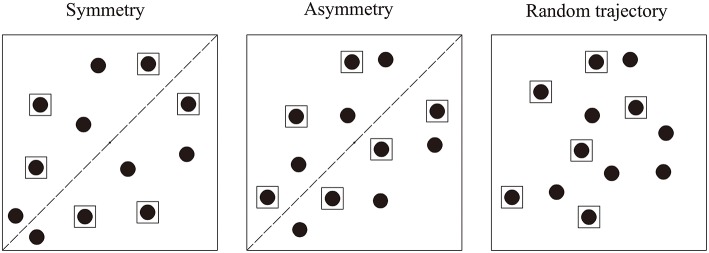
**Three target position conditions in Experiment 1: Symmetry, Asymmetry, and Random trajectory**. Disks inside the black squares were targets.

Experiment 1 was designed to investigate the automatic grouping effect of symmetry. As shown in Figure 2 (see Supplementary Videos 1–3), three grouping relations were formed based on target position: Symmetry (target-target grouping), Asymmetry (target-distractor grouping), and Random trajectory (control condition: no grouping). In addition, three conditions of target number were used: 2, 4, 6. The Random trajectory condition was used as a control to determine whether the symmetry-based grouping effect reflected facilitation in the Symmetry condition, impairment in the Asymmetry condition, or both.

According to the theory forwarded by Gestalt psychologists, regularity is a principle of perceptual grouping, so we assumed that the symmetric spatial-motion between targets would enhance inter-target grouping and then facilitate tracking performance. We also assumed that the symmetric spatial-motion between target and distractor would impair tracking performance. If symmetry-based grouping automatically guided attention, then better tracking performance in both the Symmetry condition relative to the Random trajectory condition and in the Random trajectory condition relative to the Asymmetry condition can be expected. We expected accuracy to decline as target number increases.

## Materials and methods

### Participants

A total of 31 undergraduate and graduate students (age range: 19–25 years, mean age = 23.0 ± 1.7 years) participated in Experiment 1. All participants had normal or corrected-to-normal vision. All participants gave a written consent prior to the experiment and received payment after the experiment. This study was approved by the Institutional Review Board and Ethics Committee of Human Participant Protection, School of Psychology at Beijing Normal University.

### Stimuli

Stimuli were displayed on a 17-inch CRT monitor at a resolution of 1024 × 768 pixels with a refresh rate of 85 Hz. All displays were programmed in Matlab R2013b (The Math Works) with Psychophysics Toolbox (Brainerd, 1997; Pelli, 1997). The participants used a mouse and keyboard to register their responses. Participants were seated approximately 57 cm away from the monitor; 1 cm on the screen subtends 1 degree of visual angle (°). The initial positions of the axis of symmetry and active disks were randomly chosen. The angular range of the axis was constrained to 45° from the initial position. The initial rotating direction of the axis (clockwise or counter-clockwise) was randomly chosen. The angular speed of the axis was set to 13° /s and the axis reversed direction when it reached the edges of the angular range. The speed of the active disks was randomly set to 5.5 or 7° /s. The disks bounced off the edge of the presentation window and the axis of symmetry, and they repelled one another when a minimal center-to-center distance of 1° was reached.

In each trial of the Symmetry and Asymmetry conditions, 12 disks were presented. Each disk had a symmetrically reflected disk. Therefore, six pairs of symmetrical disks (SMOT) were used. The Symmetry condition was target-target grouping based on symmetric motion. In the Symmetry condition, targets were selected from paired symmetrical disks, so each target was symmetrical with another target. Disks in each symmetrical pair either served as both targets or both distractors in the Symmetry condition. The Asymmetry condition was the target-distractor grouping based on symmetric motion. In the Asymmetry condition, only one disk from each symmetrical pair was a target. In this condition, no target was symmetrical with another target, and each target was symmetrical with a distractor. Ten abovementioned SMOT trials were ran in the background while the participants read the instructions on the screen. The computer stored trajectory of each disk of the ten trials. Because each trial had twelve disks, so a total of 120 trajectories were stored. The trajectory of each object in the Random trajectory condition was randomly selected from the 120 trajectories stored. Thus, the Random trajectory condition preserved the trajectories of SMOT, but no grouping relation was formed based on symmetry.

### Procedure

Instructions explaining the experiment were shown on the screen. The participants were asked to track the flashed objects continuously, and no mention was made of grouping or symmetry in the instruction nor in the experiment recruitment. In each trial, participants pressed the spacebar to initiate the designation period, which showed a black fixation (0.7° × 0.7°) and 12 black disks (0.5° radius) presented inside a gray presentation window (21.5° × 21.5°). Here, 2, 4, or 6 disks were flashed as targets for 2 s to avoid the uncertainty at the onset of each trial (Ma and Flombaum, 2013). In all target position conditions, the targets were randomly chosen, and the targets in the Symmetry and Asymmetry conditions were evenly distributed on both sides of the axis of symmetry. Therefore, exactly 1, 2, or 3 targets appeared on each side of the axis of symmetry. All objects then moved around the presentation window, and participants were asked to track the flashed objects. The total motion duration on a given trial randomly varied among 5, 6, and 7 s. Thereafter, the participants were required to make responses. The participants were given an unlimited time to indicate all the targets, and they were asked to guess in case they were uncertain. When a participant clicked on a disk using the mouse, a yellow frame would appear outside the disk. A selection could be cancelled by re-clicking on the disk. Participants were not informed of the results after the selection. Once the participants indicated the exact number of disks that flashed at the beginning of the trial, they could press the spacebar to initiate the next trial.

Each participant completed a total of 180 trials, i.e., 20 trials of each target position (Symmetry, Asymmetry, Random trajectory) for each target number (2, 4, 6), in random order. At the start of the experiment, five trials were provided to the participants for practice. The practice trials were randomly selected from all conditions and were excluded from the analysis. The entire experiment took approximately 50 min, with a 5 min rest after the participants completed 90 trials. All participants answered one question (“What strategy did you use to track the targets?”) over the telephone about 2 months after participating in the experiment.

### Results

**Figure 3:**
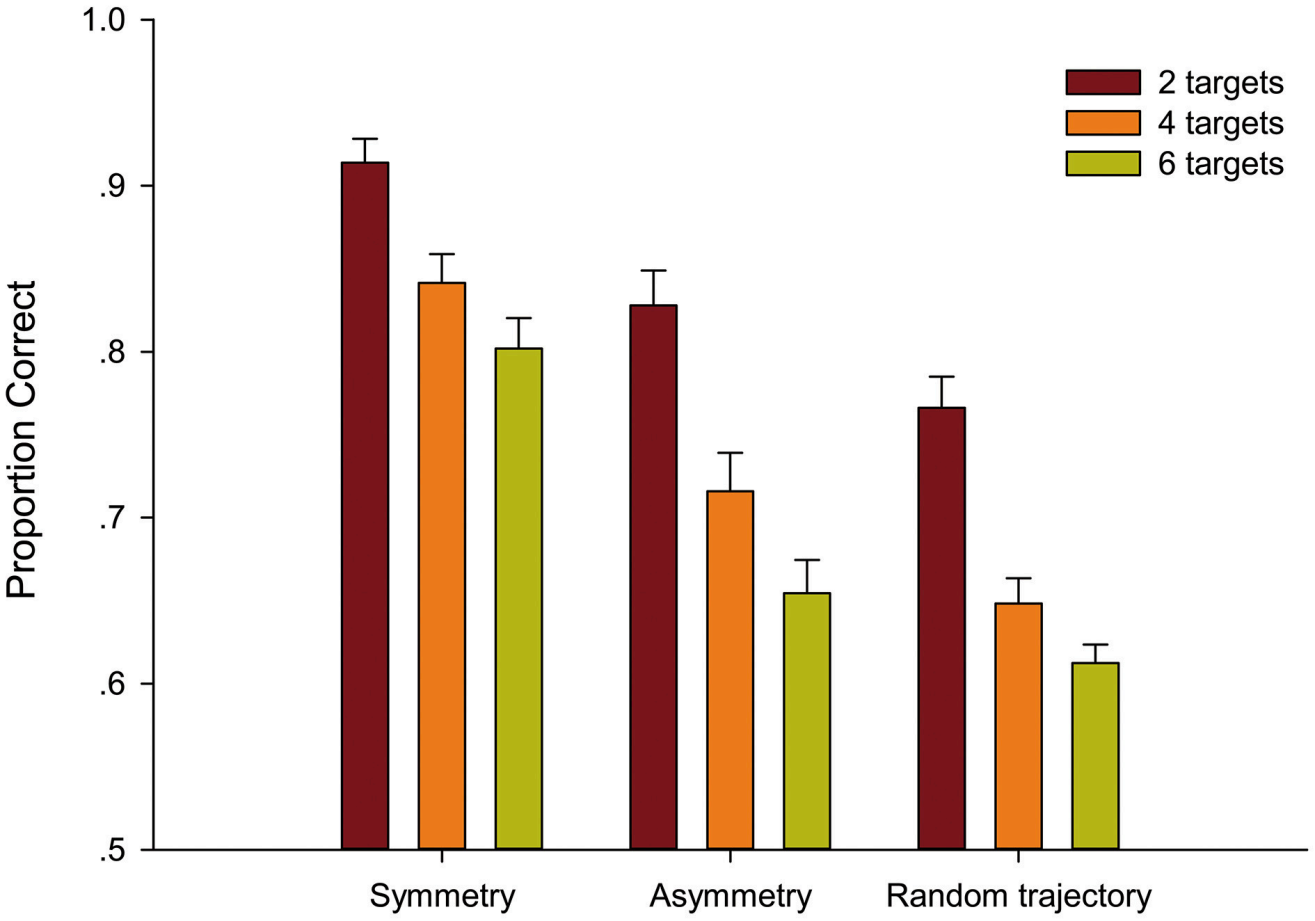
**Average proportion correct as a function of target number and target position and standard error in Experiment 1**.

The results are shown in Figure 3. The mean accuracies were submitted to a 3 (target position: Symmetry, Asymmetry, and Random trajectory) × 3 (target number: 2, 4, and 6) within-subject repeated-measures analysis of variance (ANOVA). A significant main effect of target position was observed, i.e., [*F*_(2, 60)_ = 102.06, *p* < 0.001, $\eta_{p}^{2}=$ 0.773]. Accuracy was significantly higher in the Symmetry condition (*M* = 0.85) than in the Asymmetry [(*M* = 0.73), *t*_(92)_ = 8.48, *p* < 0.001, $\eta_{p}^{2}=$ 0.439] and Random trajectory conditions [(*M* = 0.68), *t*_(92)_ = 15.83, *p* < 0.001, $\eta_{p}^{2}=$ 0.731]. The Asymmetry condition was reliably better than the Random trajectory condition [*t*_(92)_ = 4.59, *p* < 0.001, $\eta_{p}^{2}=$ 0.187]. The main effect of the target number was also significant [*F*_(2, 60)_ = 88.69, *p* < 0.001, $\eta_{p}^{2}=$ 0.747]. Accuracy was significantly higher in the 2-target condition (*M* = 0.84) than in the 4-target condition [(*M* = 0.74), *t*_(92)_ = 10.61, *p* < 0.001, $\eta_{p}^{2}=$ 0.550] and the 6-target condition [(*M* = 0.69), *t*_(92)_ = 11.58, *p* < 0.001, $\eta_{p}^{2}=$ 0.593]. The 4-target condition was reliably better than the 6-target condition [*t*_(92)_ = 4.01, *p* = 0.001, $\eta_{p}^{2}=$ 0.149]. The result clearly showed that tracking accuracy declined significantly as the number of targets increased.

The interaction between the target position and target number was significant [*F*_(4, 120)_ = 2.71, *p* = 0.033, $\eta_{p}^{2}=$ 0.083]. Simple effect analysis showed that the tracking performance of the 2-target condition was different among Symmetry, Asymmetry, and Random trajectory conditions [*F*_(2, 60)_ = 28.92, *p* < 0.001, $\eta_{p}^{2}=$ 0.493], as with the 4-target condition [*F*_(2, 60)_ = 78.28, *p* < 0.001, $\eta_{p}^{2}=$ 0.723] and the 6-target condition [*F*_(2, 60)_ = 67.68, *p* < 0.001, $\eta_{p}^{2}=$ 0.693]. The effect of target position was stronger when tracking 4 or 6 targets than 2 targets. The results revealed that when more targets were tracked, symmetry-based inter-target grouping better improved tracking performance. *Post-hoc* tests (pairwise comparison, Bonferroni corrected) within the target position and within the target number were conducted to determine that the 4-target Random trajectory condition (*M* = 0.65) was not significantly different from the 6-target Random trajectory condition [(*M* = 0.61), *t*_(30)_ = 2.29, *p* = 0.088, $\eta_{p}^{2}=$ 0.149]. The remaining comparisons were significant.

We contacted all 31 participants over the telephone and asked the strategies that they had taken. Only two participants mentioned using the regular location of paired objects as a part of their strategy to track targets. The rest of the participants never mentioned using an explicit grouping strategy.

### Discussion

The results indicated that participants utilized symmetry to group objects during tracking, and it happened without any explicit instructions. Symmetric spatial-motion between targets enhanced inter-target grouping and then facilitated tracking performance. Tracking performance of target-target grouping was significantly better than that of target-distractor grouping. The magnitude of difference between the Symmetry condition and Asymmetry condition was large, sometimes nearly 15% [6-target Symmetry condition (*M* = 0.80), 6-target Asymmetry condition (*M* = 0.65)].

In Symmetry and Asymmetry conditions, tracking accuracy all declined significantly as the number of targets increased, which was consistent with MOT tasks. In the Random trajectory condition which retained the trajectories of SMOT, tracking accuracy also declined significantly as the number of targets increased. These results showed that the trajectory of SMOT was similar as that of typical MOT, and the newly designed SMOT can be used to study object tracking. Previous experiments (Pylyshyn and Storm, 1988) have shown that observers can track a maximum of about 4 targets. In the Symmetry condition of our SMOT design, subjects were able to track 6 targets (6-target Symmetry condition (M=0.80)). This clearly showed that symmetry-based inter-target grouping can increase the target number that can be tracked. The results revealed that when more targets were to be tracked, symmetry-based inter-target grouping better improved tracking performance. The tracking accuracy of different target number (e.g., 2, 4, and 6) showed that participants can track 6 targets successfully. So the target number was fixed at 6 in the following Experiment 2, 3, and 4.

The tracking performance of the Random trajectory condition was worse than that of the Asymmetry condition. On the one hand, the worse performance in the Random trajectory condition in Experiment 1 may be attributed to trajectory overlapping and sudden change of the motion direction of objects. In the Asymmetry condition, disks repelled one another when a minimal center-to-center distance of 1° was reached. So disks in the Asymmetry condition never overlapped one another during tracking, and they changed direction only after collision. However, in the Random trajectory condition trajectories of disks were randomly selected from the stored 120 trajectories. Consequently, disks of a given trial in the Random trajectory condition didn't repel one another, instead they sometimes overlapped one another and changed direction without actual collision. On the other hand, worse performance in the Random trajectory may be because the objects were not constrained by an axis and were free to move about the screen, which would have given more opportunity for confusability of targets and distractors because this depended on their proximity during motion (Bae and Flombaum, 2012). Given the reasons mentioned above, the Random trajectory condition in Experiment 1 could not serve as a proper control and provide evidence for the automaticity of symmetry-based grouping effects.

## Experiment 2

**Figure 4:**
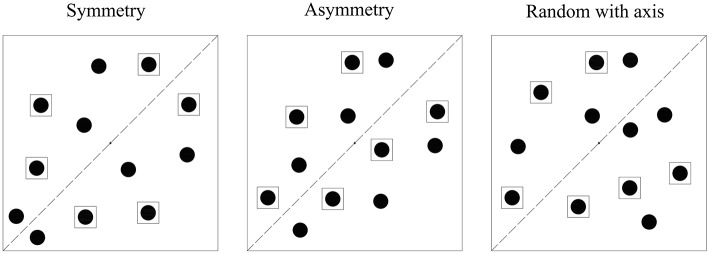
**Three target position conditions in Experiment 2: Symmetry, Asymmetry, and Random with axis**. Disks inside the black squares were targets.

The results of Experiment 1 showed that objects can be grouped based on symmetry. However, without a proper control condition, results of Experiment 1 cannot prove whether the symmetry-based grouping effects reflect facilitation in the Symmetry condition, impairment in the Asymmetry condition, or both. To provide evidence for automaticity, the main purpose of Experiment 2 was to design a new and proper control condition for the SMOT task. Three conditions of target position exist in Experiment 2: Symmetry, Asymmetry, and Random with axis (Figure 4). The Symmetry and Asymmetry conditions were the same as those of Experiment 1. In the Random with axis condition, an invisible axis existed, which was a replication of the axis of symmetry of the SMOT (see Supplementary Video 4). This axis retained all motion parameters of the axis of symmetry. Six disks were on each side of the invisible axis in the Random with axis condition. Unlike SMOT, where disks on only one side of the axis of symmetry were active, disks on both sides of the axis were active in the Random with axis condition. Consequently, in the latter condition, disks on one side of the axis moved independently from disks on the other side of the axis, and the symmetric relation no longer paired disks on the two sides of the axis. Objects in the Random with axis condition were constrained by an axis just like in the Symmetry and Asymmetry conditions. In addition, no trajectory overlapping existed, and sudden change of object motion direction occurred in the Random with axis condition.

## Materials and methods

### Participants

A total of 24 undergraduate and graduate students (age range: 21–28 years, mean age = 24.5 ± 2.0 years) participated in this experiment. All reported normal or corrected-to-normal vision. The students did not participate in Experiment 1.

### Stimuli

A total of 12 disks were presented and 6 disks were flashed as targets for 2 s. Targets were randomly chosen and evenly distributed on both sides of the axis, i.e., 3 targets on each side. Three grouping relations existed based on target position. The Symmetry and Asymmetry conditions were identical to those in Experiment 1. In the Random with axis condition, the parameters of the axis were the same as those in Experiment 1, and the motions of disks on both sides of the axis were the same as the motions of the active disks in the Symmetry or Asymmetry conditions. Six disks existed on each side of the axis, and the disks never crossed the invisible axis. The disks in the Random with axis condition bounced off the edge of the presentation window and the invisible axis, and repelled one another when a minimal center-to-center distance of 1° was reached.

### Procedure

The procedure followed for Experiment 2 was the same as that for Experiment 1. Each participant completed 60 trials, with 20 trials of each target position (Symmetry, Asymmetry, Random with axis) in random order. The experiment was completed within approximately 20 min without time for rest. Upon completion of the entire experiment, participants were asked to answer the following question: “What strategy did you use to track the targets?”

### Results

**Figure 5:**
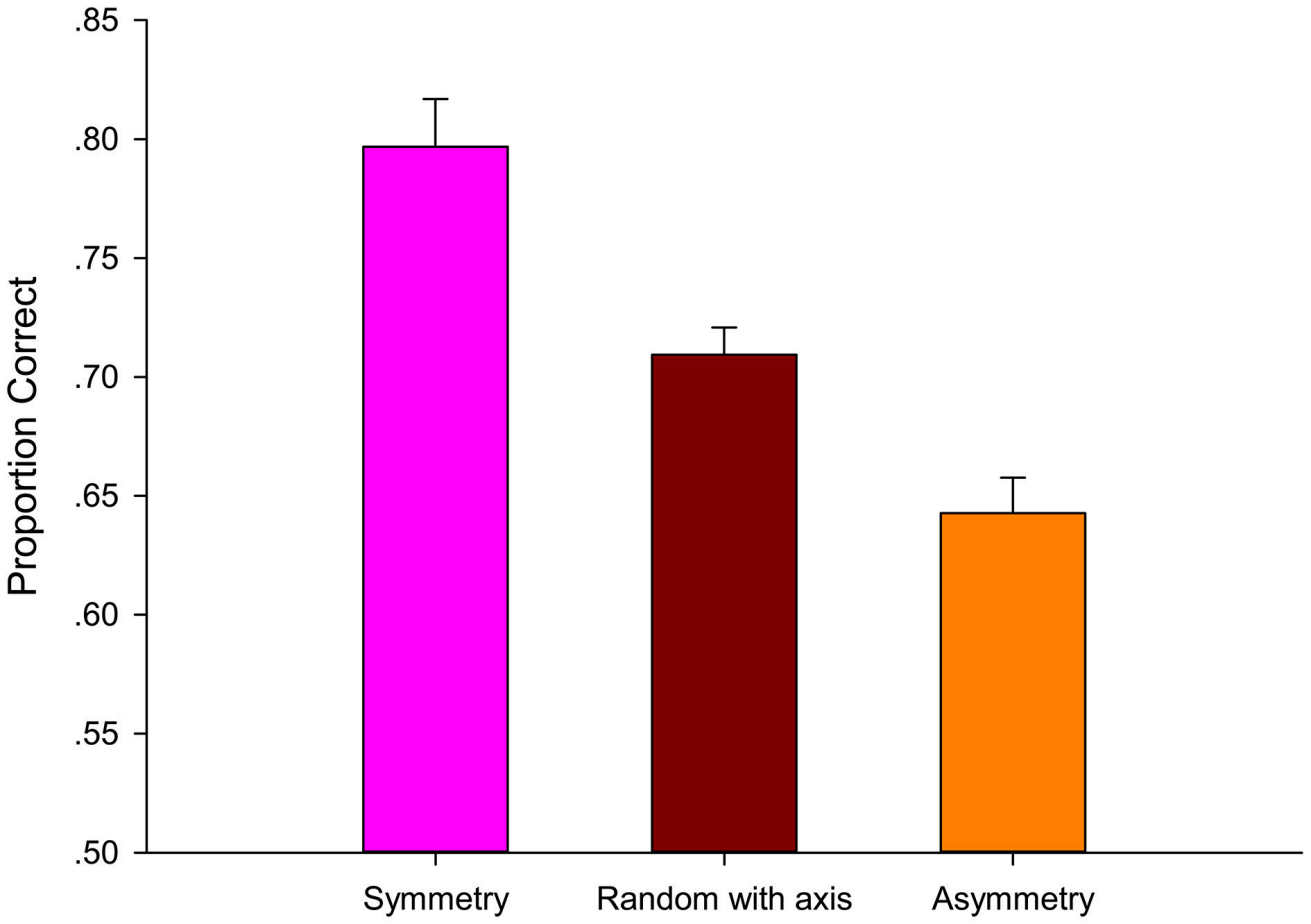
**Average tracking performance as a function of target position and standard error in Experiment 2**.

Results are shown in Figure 5. The mean accuracies were submitted to a 3 (target positions: Symmetry, Asymmetry, and Random with axis) within-subject repeated-measures ANOVA. A significant main effect of target position existed [*F*_(2, 46)_ = 36.56, *p* < 0.001, $\eta_{p}^{2}=$ 0.614]. Follow-up pairwise comparisons showed that the Symmetry condition (*M* = 0.80) yielded higher tracking accuracy than the Asymmetry condition [(*M* = 0.64), *t*_(23)_ = 6.57, *p* < 0.001, $\eta_{p}^{2}=$ 0.652], and Random with axis condition [(*M* = 0.71), *t*_(23)_ = 5.48, *p* < 0.001, $\eta_{p}^{2}=$ 0.566]. The Random with axis condition was better than the Asymmetry condition [*t*_(23)_ = 5.02, *p* < 0.001, $\eta_{p}^{2}=$ 0.523].

All participants answered the question about what strategy they had taken after Experiment 2. Only three participants mentioned using regular location of paired objects as part of their strategy to track targets. The remaining participants never mentioned using an explicit grouping strategy.

### Discussion

Experiment 2 replicated the symmetry-based grouping effect on tracking performance found in Experiment 1. Furthermore, by comparing the Symmetry condition to the Random with axis condition, and the Random with axis condition to the Asymmetry condition, we found evidence for automatic symmetry-based grouping. Performance was significantly improved when targets were in a symmetrical relation relative to when no grouping relation based on symmetry existed. In addition, performance was inferior when targets and distractors were in a symmetrical relation relative to when no grouping relation based on symmetry existed. This symmetry-based grouping was automatic because it occurred even when it was consistently detrimental to performance and irrelevant to task instructions. Results of Experiment 2 demonstrated that participants automatically adopted symmetrical spatial-motion information for grouping during tracking.

## Experiment 3

In Experiment 3, we investigated whether the grouping effects based on symmetry and feature similarity were additive. Different principles of grouping can cooperate or compete with one another (Lier and Wagemans, 1997). In Experiment 3, we intended to study the cooperation and competition of two different grouping principles: regularity and similarity. Although the feature-based groupings did not seem to be additive (Erlikhman et al., 2013), grouping based on different principles such as symmetry-regularity and feature-similarity would possibly produce an additive grouping effect. In Experiment 3, there were two grouping relations based on symmetry: Symmetry (target-target grouping based on symmetry) and Asymmetry (target-distractor grouping based on symmetry); and three grouping relations based on color: Identical (no grouping relation), T-T (target-target grouping based on color), and T-D (target-distractor grouping based on color). To provide evidence for additivity, we compared the T-T Symmetry condition with the Identical Symmetry condition and with the T-T Asymmetry condition. The T-T Symmetry condition was the target-target grouping of color and symmetry, and the Identical Symmetry and the T-T Asymmetry conditions were the target-target grouping of single symmetry and single color, respectively. Higher accuracy in the T-T Symmetry condition relative to the Identical Symmetry condition and to the T-T Asymmetry condition indicated that the grouping of symmetry and color showed greater improved performance than the grouping of sole symmetry and grouping of sole color. Moreover, this higher accuracy would be ascribed to the additivity of symmetry-based and color-based grouping effects.

## Materials and methods

### Participants

A total of 25 undergraduate and graduate students (age range: 19–26 years, mean age = 23.1 ± 1.7 years) participated in Experiment 3. All reported normal or corrected-to-normal vision. The students did not participate in Experiments 1 and 2.

### Stimuli

A total of 12 colored disks were used, and 6 of them were highlighted as targets in yellow boxes [1° × 1°, RGB (255,255,0)] for 2 s. The colors of the disks were chosen randomly from green [RGB (0,255,0)], magenta [RGB (255,0,255)], cyan [RGB (0,255,255)], blue [RGB (0,0,255)], red [RGB (255,0,0)], orange [RGB (255,165,0)], and deep pink [RGB (255,20,147)] according to a trial's condition. A total of seven different colors were used, because previous studies (Pinto et al., 2010) showed that using repeated color from trial to trial improves tracking performance. In all trials of Experiment 3, all disks turned black [RGB (0,0,0)] 300 ms before the end of the movement. The remaining stimuli were the same as in Experiment 1.

### Procedure

**Figure 6:**
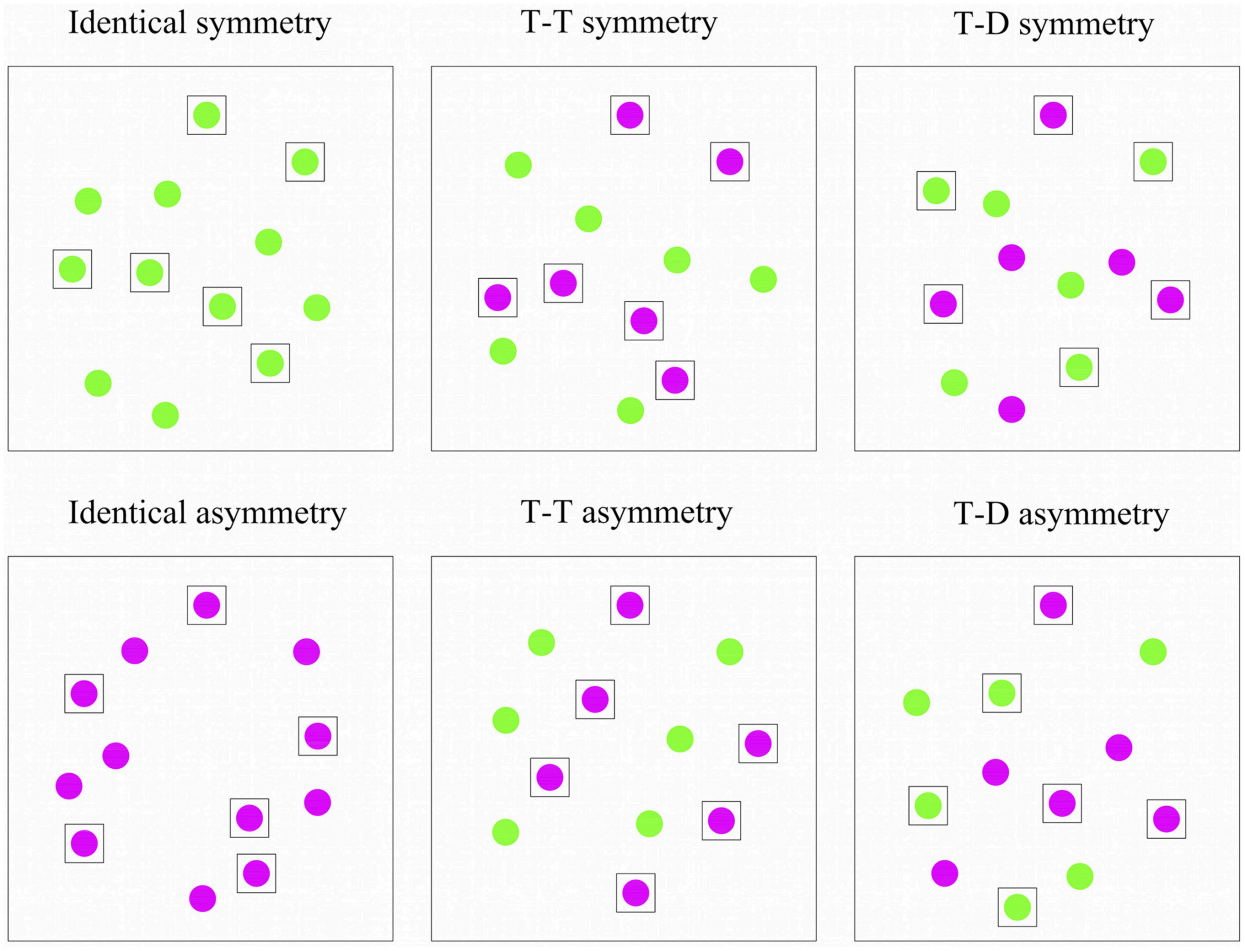
**Stimuli for Experiment 3**. Six conditions classified by the grouping relation based on symmetry and color. Disks inside the black squares were targets.

As shown in Figure 6, two grouping relations existed based on symmetry: Symmetry (target-target grouping based on symmetry) and Asymmetry (target-distractor grouping based on symmetry); and three grouping relations based on color: Identical (no grouping relation), T-T (target-target grouping based on color), and T-D (target-distractor grouping based on color). The Symmetry and Asymmetry conditions were the same as those in Experiment 1. In the Identical condition, all disks always shared the same color. In the T-T condition, all targets shared a single color, whereas all the distractors shared another color. In the T-D condition, three targets and three distractors shared a single color and the remaining objects shared another color (see Supplementary Videos 5–10).

The procedure followed for Experiment 3 was the same as that for Experiment 1. Each participant completed 120 trials, 20 trials of each symmetry relation (Symmetry, Asymmetry) for each color relation (Identical, T-T and T-D) in random order. The experiment was completed within approximately 35 min without time for rest. All participants answered the question: “What strategy did you use to track the targets?” over the telephone approximately 2 months after participation in the experiment.

### Results

**Figure 7:**
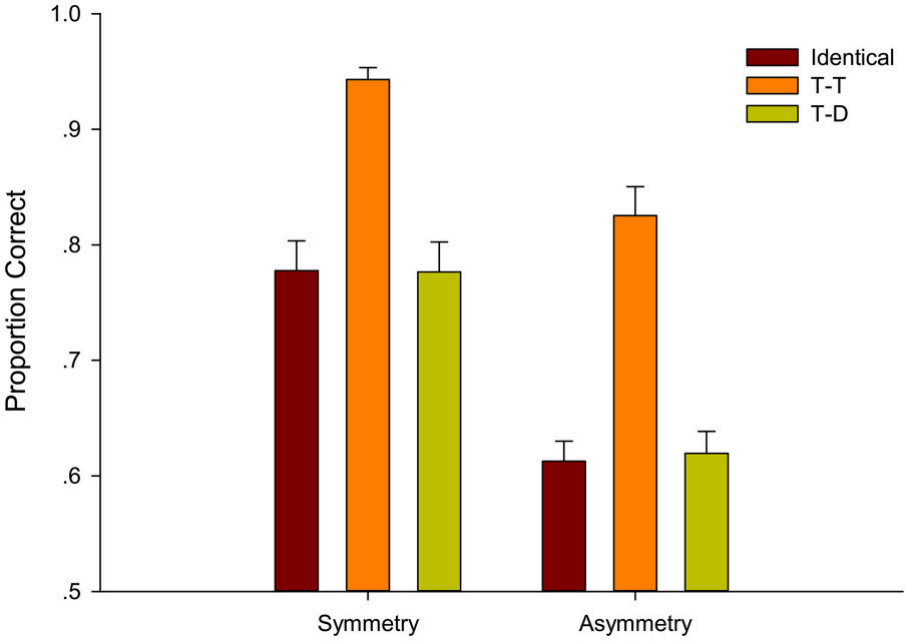
**Average proportion correct and standard error for six conditions classified by groupings based on symmetry and color in Experiment 3**.

Descriptive results are presented in Figure 7. The mean accuracies were submitted to a 2 (symmetry-based grouping: Symmetry, Asymmetry) × 3 (color-based grouping: Identical, T-T, and T-D) within-subject repeated-measures ANOVA. A significant main effect of symmetry-based groupings existed [*F*_(1, 24)_ = 33.419, *p* < 0.001, $\eta_{p}^{2}=$ 0.582]. The accuracy in the Symmetry condition (*M* = 0.83) was significantly better than that in the Asymmetry condition (*M* = 0.69). The main effect of color-based grouping was also significant [*F*_(1, 33)_ = 131.040, *p* < 0.001, $\eta_{p}^{2}=$ 0.845], thereby revealing the grouping effect of color. Follow-up pairwise comparisons showed that accuracy in the T-T condition (*M* = 0.88) was significantly better than those in the Identical condition [(*M* = 0.70), *t*_(49)_ = 12.285, *p* < 0.001, $\eta_{p}^{2}=$ 0.755] and T-D condition [(*M* = 0.70), *t*_(49)_ = 11.970, *p* < 0.001, $\eta_{p}^{2}=$ 0.745]. The T-D condition was not reliably better than the Identical condition [*t*_(49)_ = 0.377, *p* = 1, $\eta_{p}^{2}=$ 0.003]. In addition, the interaction between the target position and color was not significant [*F*_(2, 38)_ = 2.943, *p* = 0.076, $\eta_{p}^{2}=$ 0.109].

Pairwise comparison (Bonferroni corrected) within symmetry-based grouping relations and within color-based grouping relations were conducted to find that the T-T Symmetry condition (*M* = 0.94) yielded higher tracking accuracy than the Identical Symmetry condition [(*M* = 0.78), *t*_(24)_ = 8.41, *p* < 0.001, $\eta_{p}^{2}=$ 0.747] and the T-T Asymmetry condition [(*M* = 0.83), *t*_(24)_ = 4.71, *p* < 0.001, $\eta_{p}^{2}=$ 0.480]. The T-T Symmetry condition was the target-target grouping of color and symmetry, and the Identical Symmetry condition and the T-T Asymmetry condition were the target-target grouping of single symmetry and single color, respectively.

The abovementioned results demonstrated that the grouping of symmetry and color improved performance better than the grouping of sole symmetry and grouping of sole color; thus, groupings based on symmetry and color were additive. The combination of symmetry and color had stronger facilitation than sole symmetry and sole color on grouping effect in MOT tasks. The Identical Symmetry condition (*M* = 0.78) and the T-D Symmetry condition (*M* = 0.78) were not significantly different [*t*_(24)_ = 0.09, *p* = 1, $\eta_{p}^{2}=$ 0.0003], along with the Identical Asymmetry condition (*M* = 0.61) and the T-D Asymmetry condition [(*M* = 0.62), *t*_(24)_ = –0.76, *p* = 1, $\eta_{p}^{2}=$ 0.024]. We performed paired samples *T*-test and found that the T-D Symmetry condition (*M* = 0.78) and the T-T Asymmetry condition (*M* = 0.83) were not significantly different [t_(24)_ = –1.288, *p* = 0.210], indicating that symmetry and color were equally strong when they competed with each other by supporting different groupings.

We contacted all participants over telephone and asked what strategies they had taken. Only two mentioned using the regular location of paired objects as part of their strategies to track targets. The remaining participants never mentioned using an explicit grouping strategy.

### Discussion

Symmetric motion as spatial-motion information enhanced the grouping effect, and its combination with the feature-based grouping of color proved to be additive. The results showed that the tracking performances all significantly improved when adding target-target grouping of color with the target-target grouping of symmetric motion or adding target-target grouping of symmetric motion with the target-target grouping of color. The results indicated that spatial symmetry-based grouping and feature-based grouping of color were additive.

The relative strengths of color and symmetry grouping cues can be interpreted by comparing the T-T Asymmetry condition and the T-D Symmetry condition. The T-T Asymmetry condition was not significantly better than the T-D Symmetry condition, which indicated that color grouping and symmetry grouping were equally strong.

Using color and symmetry in Experiment 3, we proved the existence of an additivity grouping effects of regularity and similarity in SMOT tasks. We used another commonly used feature to test this additivity of grouping effects in order to investigate whether the additive grouping effect was stable. In Experiment 4, we manipulated shape as the surface feature in the SMOT task, and intended to further investigate the additive grouping effect of symmetry and similarity.

## Experiment 4

## Materials and methods

### Participants

A total of 25 undergraduate and graduate students (age range: 19–26 years, mean age = 22.2 ± 1.9 years) participated in this experiment. All reported normal or corrected-to-normal vision. The students did not participate in Experiments 1, 2, and 3.

### Stimuli

**Figure 8:**
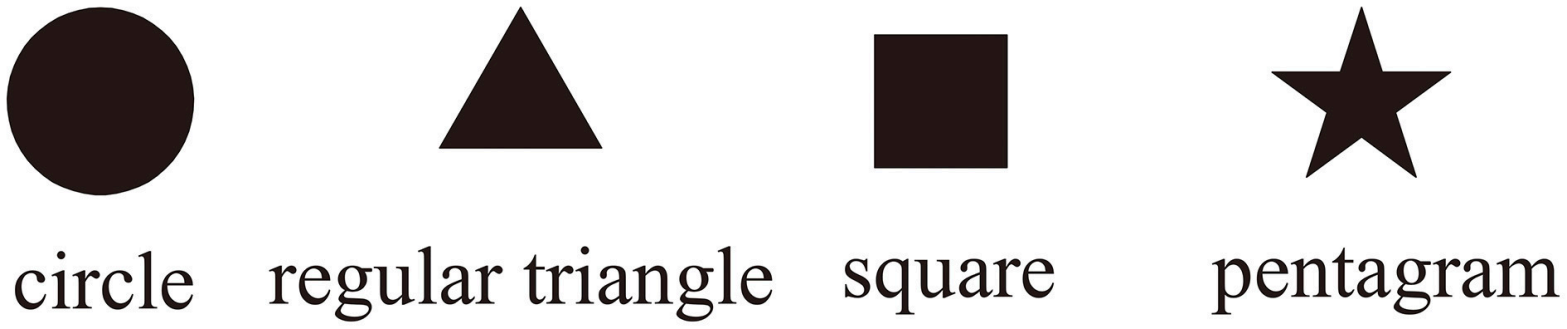
**Stimuli used in Experiment 4**.

A total of 12 shape objects were used, and 6 of them were highlighted as targets in yellow boxes [1° × 1°, RGB (255,255,0)] for 2 s. The shapes we used in Experiment 4 were circle (0.5° radius), regular triangle, square, and pentagram (Figure 8). The triangle and square were inscribed geometric drawings of the circle, and the pentagram was drawn from inscribed regular pentagon of the circle. All shapes were black [RGB (0,0,0)]. In all trials of Experiment 4, all shape objects were transformed into identical disks 300 ms before the end of the movement. The rest of the stimuli were the same as in Experiment 1.

### Procedure

Two grouping relations existed based on symmetry: Symmetry (target-target grouping based on symmetry) and Asymmetry (target-distractor grouping based on symmetry). Three grouping relations were designed based on shape: Identical (no grouping relation), T-T (target-target grouping based on shape), and T-D (target-distractor grouping based on shape). In the Identical condition, all objects shared the same shape. In the T-T condition, all targets shared a single shape, whereas all distractors shared another shape. In the T-D condition, three targets and three distractors shared a single shape, and the remaining objects shared another shape.

The procedure followed for Experiment 4 was the same as that for Experiment 1. Each participant completed 120 trials, with 20 trials of each symmetry-based grouping (Symmetry, Asymmetry) for each shape-based grouping (Identical, T-T, and T-D) in random order. The experiment was completed within approximately 35 min without time for rest. All of the participants answered the question: “What strategy did you use to track the targets?” over the telephone approximately 2 months after participation in the experiment.

### Results

**Figure 9:**
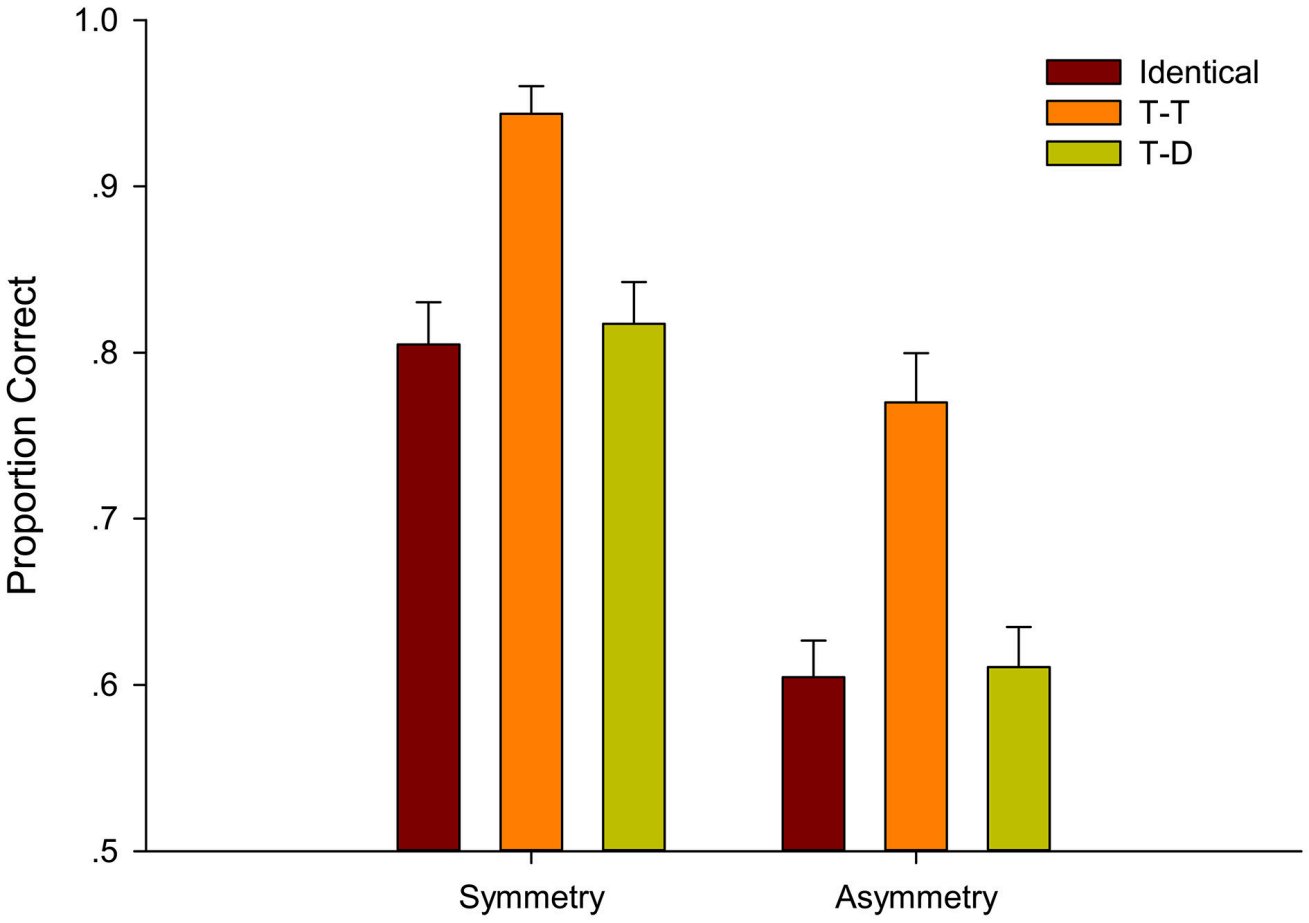
**Average proportion correct and standard error for six conditions classified by groupings based on symmetry and shape in Experiment 4**.

The descriptive results are shown in Figure 9. The mean accuracies were submitted to a 2 (symmetry-based grouping: Symmetry, Asymmetry) × 3 (shape-based grouping: Identical, T-T, and T-D) within-subject repeated-measures ANOVA. A significant main effect of symmetry-based grouping existed [*F*_(1, 24)_ = 44.872, *p* < 0.001, $\eta_{p}^{2}=$ 0.652]. The accuracy in the Symmetry condition (*M* = 0.86) was better than that in the Asymmetry condition (*M* = 0.66). The main effect of shape-based grouping was also significant [*F*_(2, 48)_ = 78.628, *p* < 0.001, $\eta_{p}^{2}=$ 0.766]. Follow-up pairwise comparisons showed that accuracy in the T-T condition (*M* = 0.86) was significantly better than both the Identical condition [(*M* = 0.70), *t*_(49)_ = 10.01, *p* < 0.001, $\eta_{p}^{2}=$ 0.67] and the T-D condition [(*M* = 0.71), *t*_(49)_ = 8.82, *p* < 0.001, $\eta_{p}^{2}=$ 0.61]. The T-D condition was not reliably better than the Identical condition [*t*_(49)_ = 1.18, *p* = 1, $\eta_{p}^{2}=$ 0.030]. No interaction was found, *p* = 0.225, $\eta_{p}^{2}=$ 0.060.

Pairwise comparison (Bonferroni corrected) within target position and within shape were conducted and found that the T-T Symmetry condition (*M* = 0.94) yielded higher tracking accuracy than the Identical Symmetry condition [(*M* = 0.80), *t*_(24)_ = 8.37, *p* < 0.001, $\eta_{p}^{2}=$ 0.745] and the T-T Asymmetry condition [(*M* = 0.77), *t*_(24)_ = 5.78, *p* < 0.001, $\eta_{p}^{2}=$ 0.582]. The T-T Symmetry condition was the target-target grouping of shape and symmetry, and the Identical Symmetry condition and the T-T asymmetry condition were the target-target grouping of single symmetry and single shape, respectively.

The abovementioned results showed that the combination of shape and symmetric motion grouping was better than sole symmetry grouping and sole shape grouping were. Consequently, groupings of shape and symmetry were additive. The Identical Symmetry condition (*M* = 0.80) and T-D Symmetry condition (*M* = 0.82) were not significantly different [*t*_(24)_ = −1.03, *p* = 0.936, $\eta_{p}^{2}=$ 0.043] as were the Identical Asymmetry condition (*M* = 0.60) and T-D Asymmetry condition [(*M* = 0.61), *t*_(24)_ = −0.88, *p* = 1, $\eta_{p}^{2}=$ 0.031]. We performed paired samples *T* test and found that the T-D Symmetry condition (*M* = 0.82) and the T-T Asymmetry condition (*M* = 0.77) were not significantly different [t_(24)_ = 1.204, *p* = 0.240]. All these results were consistent with Experiment 3.

We contacted all 25 participants over telephone, and asked what strategies they had taken. Only one mentioned using regular location of paired objects as a part of a strategy to track targets. The remaining participants never mentioned using an explicit grouping strategy.

### Discussion

Experiment 4 was similar to Experiment 3, except that the grouping in the former was shape-based. Again, the results of Experiment 4 showed that symmetry-based grouping enhanced tracking performance and that grouping based on symmetry and shape facilitated tracking more than that based on only one cue (e.g., symmetry). Taken together, the findings of Experiments 3 and 4 provided evidence for a possible additivity of grouping effects when grouping was based on two different principles.

## General discussion

Grouping based on feature similarity affects tracking performance in MOT tasks (Makovski and Jiang, 2009a,b; Feria, 2012; Howe and Holcombe, 2012; Erlikhman et al., 2013). In addition, feature-based grouping effects do not seem to be additive (Erlikhman et al., 2013). One problem with the previous studies on grouping effects in MOT is that grouping occurs based on feature similarity alone (Makovski and Jiang, 2009b; Feria, 2012; Erlikhman et al., 2013). Gestalt psychologists have proposed several principles of grouping. When discussing the additivity of grouping effects in MOT, including other types of grouping cues is better. Grouping effects based on different perceptual grouping principles may be additive. Previous feature-based groupings showed that when two different shapes were added to a condition where targets and distractors already had two different colors, performance was not improved compared with the previous condition (Erlikhman et al., 2013). They compared a condition where targets and distractors had different shapes, colors and sizes with a condition where targets and distractors only had different colors, and found no difference between these two conditions. We compared a condition (i.e., T-T Symmetry) where targets were in symmetric relation and had the same color (different from distractors' color) with a condition (i.e., T-T Asymmetry) where targets and distractors only had different colors (also with a condition where targets were only in symmetric relation, i.e., Identical Symmetry), and found a significantly better tracking performance in the former condition. Our results showed that symmetry-based and feature-based groupings are additive.

The current study provides spatiotemporal regularity properties (e.g., symmetrical motion) and object feature similarity for participants to group objects during tracking. We attempt to investigate whether additive grouping effects exist based on symmetry and feature similarity. The results of Experiments 1 and 2 revealed the automatic symmetry-based grouping. The grouping effect based on symmetry showed that spatial-motion information could be used automatically by participants. Experiments 3 and 4 provide evidence for the additivity of grouping effects of symmetry-based and feature-based (color or shape) groupings.

In the questions that we collected after each experiment, very few participants reported using an explicit grouping strategy. A total of 2 (out of 31) participants in Experiment 1, 3 (out of 24) in Experiment 2, 2 (out of 25) in Experiment 3, and 1 (out of 25) in Experiment 4 mentioned using regular motion of paired objects as a part of a strategy to track targets. This shows that participants were not using an explicit strategy to use symmetry to group and track the objects.

As far as we know, this is the first paper to determine the additive grouping effects of symmetry-based and feature-based groupings. Three possible explanations exist for the additivity of grouping effects. First, the additive grouping effects may occur in the condition where grouping is based on two different perceptual grouping principles (e.g., similarity and regularity). Grouping based on symmetry is governed by the principle of regularity, and grouping based on color/shape is governed by the principle of similarity. Many previous studies have focused only on groupings based on the principle of similarity (Makovski and Jiang, 2009b; Erlikhman et al., 2013). The feature-based grouping effect is based on the similarity of physical surface features. Similarity information of physical surface features is used as distinguishing information to group targets and improve the tracking performance. If similarity is used only as distinguishing information, then single feature difference has reached the distinguishing goal. This distinguishing effect can approximately be understood, similar to binary code in computer processing. Of course, differences exist in the degree of differentiation between different features. Tracking performance cannot be improved if we superimposed another different feature on targets and distractors, which are already distinguishable by one feature.

Second, the additive grouping effect in the present study was based on two different types of visual information, spatial-motion, and surface feature, which have different pathways into the visual system. Consequently, the symmetry-based and feature-based groupings do not compete but can be used together. Ungerleider and Mishkin (1982) suggested that based on both the anatomical and functional evidence, two pathways exist in visual processing: a dorsal pathway from V1 to the posterior parietal cortex, including the middle temporal area, which is concerned with localizing where objects are; and a ventral pathway extending from V1 to the inferior temporal cortex, including area V4, which identifies what the objects are. A previous study confirmed the two functional pathways of ventral (object) and dorsal (spatial) in humans (Haxby et al., 1994). According to the study, the spatial-motion (“where”) information and feature (“what”) information are used independently through two different pathways by the visual system to facilitate tracking performance. Hence, the additivity of grouping was found in our study. The feature information and motion information can both contribute to tracking performance independently. However, two feature-based groupings did not seem additive in previous studies (Makovski and Jiang, 2009b; Erlikhman et al., 2013), because they have the same pathway into the visual system. Two feature-based groupings would inevitably compete for resources. The present study further proves that feature information and spatial-motion information have independent processing channels.

Third, feature-based groupings that did not seem to be additive can be explained by Feria's second hypothesis: top-down setting treats the conjunction of the targets' features as a whole, and that distractors would attract attention only when they had exact same features of the targets (Feria, 2012). Hence, distractors sharing a single distinct feature with the targets would be treated equally with distractors that had two distinct features with the targets, and their tracking performance would be equal. However, symmetry offered spatial-motion based information that cannot be regarded as a whole with feature information by top-down settings, and is adopted and combined together with features to produce an additive grouping effects in the present study.

The T-D grouping based on feature (color or shape) does not impair tracking performance compared with the Identical condition. The previous findings have shown that the trend of lower accuracy in the target-distractor grouping condition in comparison to the Identical condition was unstable (Makovski and Jiang, 2009b). A high similarity between distractors and targets interferes with tracking performance even when they were in the unattended regions (Störmer et al., 2011). Erlikhman et al. found that performance in the target-distractor grouping condition was significantly worse than in the homogeneous condition. However, Makovski and Jiang (2009b) found impairment in one of their experiments but not in the other experiment. In addition, in our study the impairment was not found in either the target Symmetry or the target Asymmetry condition. Thus, further research is needed to discuss this unstable trend. The additive grouping effect of feature-based and spatial-motion-based information makes us wonder whether groupings based on any two different kinds of principles can be additive or not. What determines the additivity of grouping effect? These questions need further discussion in follow-up studies.

In conclusion, the present study demonstrates that spatial-motion symmetry can be used automatically for grouping. More importantly, we find the additivity of the grouping effects of symmetry and color (or shape) in SMOT. Our findings indicate that the grouping effects can be based on spatiotemporal properties, such as symmetry, and that grouping effects can be additive based on feature similarity and symmetry regularity. Our results imply that spatial-motion information can be used together with physical surface feature information to guide attention during tracking, which may be related to the different pathways of “where” and “what”.

## Author contributions

CW, XZ made major contributions to the design of the experiment, the interpretation of data and drafting of the article. YL, CL were responsible for offering critical comments of the design, data acquisition and analysis and revising the manuscript.

## Funding

This work was supported by the National Natural Science Foundation of China (31271083) and the National Basic Research program of China (2011CB711000).

### Conflict of interest statement

The authors declare that the research was conducted in the absence of any commercial or financial relationships that could be construed as a potential conflict of interest. The reviewer, MC and handling Editor declared their shared affiliation, and the handling Editor states that the process nevertheless met the standards of a fair and objective review.
